# Dual-Quaternion Analytic LQR Control Design for Spacecraft Proximity Operations

**DOI:** 10.3390/s21113597

**Published:** 2021-05-21

**Authors:** Kyl Stanfield, Ahmad Bani Younes

**Affiliations:** Department of Aerospace Engineering, San Diego State University, 5500 Campanile Drive, San Diego, CA 92182-1308, USA; abaniyounes@sdsu.edu

**Keywords:** dual-quaternion, dynamics, linearization, control, linear quadratic, proximity operation

## Abstract

Proximity operations offer aggregate capability for a spacecraft operating in close proximity to another spacecraft, to perform on-orbit satellite servicing, or to a space object to perform debris removal. To utilize a spacecraft performing such advanced maneuvering operations and perceiving of the relative motion of a foreign spacecraft, these trajectories must be modeled accurately based on the coupled translational and rotational dynamics models. This paper presents work towards exploiting the dual-quaternion representations of spacecraft relative dynamics for proximity operations and developing a sub-optimal control law for efficient and robust maneuvers. A linearized model using dual-quaternions for the proximity operation was obtained, and its stability was verified using Monte Carlo simulations for the linear quadratic regulator solution. A sub-optimal control law using generalized higher order feedback gains in dual-quaternion form was developed based on small error approximations for the proximity operation and also verified through Monte Carlo simulations. Necessary information needed to understand the theory behind the use of the dual-quaternion is also overviewed within this paper, including the validity of using the dual-quaternions against their Cartesian or quaternion equivalents.

## 1. Introduction

Spacecraft missions regularly require six-degree-of-freedom (DOF) maneuvers— translation and reorientation—subject to various constraints such as mechanical dynamics, environmental effects, and design constraints. High precision and robustness for spacecraft maneuvers are essential demands to achieve successful and efficient missions. Different methods have been introduced to describe the dynamical models for the 6 DOF satellite maneuvers, including relative motions. The translational dynamics are given generally in Cartesian coordinates, as in Cowell’s formulations [1]. The attitude of a spacecraft defines its orientation with respect to a reference frame [2]. The relative orientation of an estimated attitude with respect to the true attitude defines the attitude error [3,4,5]. The attitude kinematics describe the instantaneous orientation of a rigid body relative to some reference frame [6]. The kinetic and kinematic equations predict the future translational and orientational state of the spacecraft by using dynamical models to extrapolate the system state history. This methodology reveals a promising solution that will help provide an elegant and yet sub-optimal formulation of the coupled 6 DOF spacecraft maneuvers in proximity operations.

This paper presents compact nonlinear dual-quaternion kinetic and kinematic equations that can be used in control and/or estimation dynamics problems. The use of dual-quaternions provides a compact solution to represent both the orientation and position of a rigid body. The rise in the dual-quaternion’s usage is imbued in the fact that it can combine the translational and rotational kinematic and dynamic equations of motion. Moreover, control laws based on the dual-quaternion involving combined position and attitude intrinsically account for the natural coupling between the rotational and translational motion [7]. In robotics, the dual-quaternion has been used to formulate dynamic constraints for articulated robotic systems [8] or to calculate relative orientation [9]. In the field of aerospace engineering, the dual-quaternion has been investigated for a wide array of purposes—many involving relative motion [10], dynamics [11], and methods of kinematic control [12] or estimation [13] for a rigid body. Specifically, the dual-quaternion has been investigated for the use of powered descent guidance, as well as optimal powered descent guidance [14]. Dual-quaternion usage and research in the field of aerospace engineering also includes the areas of dual-quaternion-based spacecraft rendezvous [15], as well as controllers for satellite proximity operations [16]. All of these take advantage of the dual-quaternion’s properties, which combine the translational and rotational equations of motion into single equations, therefore allowing control laws that account for the natural coupling between them [17].

The proximity operation is an area of particular interest. In-orbit servicing missions have been an increasing desire for many involved in the satellite industry, and such a task would require two (or more) satellites flying in close proximity relative to one another [18] or missions targeting uncooperative space objects [19]. One of the greatest challenges for this task exists in the modeling, control, and estimation. Extreme precision is required to control not only one satellite’s relative attitude, but also its relative position. As a result, an approach that combines the coupled motions is preferable.

For proximity operation dynamics, the small relative error allows acceptable linearization of the state dynamics. As such, our approach linearizes the state dynamics in dual-quaternion (DQ) form and adopts the use of an optimal linear quadratic regulator (LQR) controller for the linearized DQ equations. The calculated control gains are then verified for global stability using the nonlinear DQ dynamics. A similar approach has been used with spacecraft control systems based on the quaternion model [20], whereas this paper focused on the dual-quaternion model. Additionally, a generalized higher order “sub-optimal” feedback control solution was developed for the nonlinear dual-quaternions of the proximity operation model.

The key contribution of this paper was developing a sub-optimal feedback control for the 6 DOF spacecraft dynamics proximity operation represented with the dual-quaternion model. The control solution was designed by linearizing the full dual-quaternion dynamics about a nominal steady-state equilibrium and obtaining reference optimal gains. The linearized control design was embodied in a framework that integrated the full nonlinear dual-quaternion dynamics to provide a robust and sub-optimal control solution for the spacecraft proximity operation. This methodology revealed a promising solution that will help provide an elegant and yet sub-optimal formulation of the coupled 6 DOF spacecraft maneuvers in proximity operations. All necessary details are clearly presented in the paper, including the theory that builds up the use of the dual-quaternion and the validation of its equations. The fundamental contributions of this paper are summarized below:

- The development of a linearized compact form for the full nonlinear dual-quaternion dynamics of the spacecraft 6 DOF maneuvers. The linearized dynamics are formulated based on small error approximations for the proximity operations;
- Verification and analysis of the stability of the linearized dual-quaternions dynamics using Monte Carlo simulations from arbitrary departure points with LQR feedback control input;
- The development of a dual-quaternion linearized-based control framework that exploits the local stability of the linearized form and implements it in the full nonlinear dynamics;
- The the new linear dynamics establish a new direction to investigate the feedback gain parameter uncertainties for the system while also providing the advantages of simplifying the complex 6 DOF dynamics into a form for optimal control that can manage system perturbations;
- Formulating tensor-based, polynomial-based, nonlinear feedback control gains equations that generalize the feedback dynamics to provide a rigorous technique to handle system nonlinearity effects and disturbances.

## 2. Mathematical Preliminaries

It is first worthwhile to overview some of the fundamentals that are used within the theory and formulations that help develop the mathematical models discussed within this paper. This section discusses only mathematical preliminaries that build up to using the dual-quaternions in the context of this paper and may be considered as either a starting point of reference or a review of information. The mathematical equivalencies between the Cartesian, quarternion, and dual-quaternion models for spacecraft control have been demonstrated previously [21], so this focuses on only the necessary background.

### 2.1. Quaternion

Quaternions are an application of complex numbers to $R^{4}$ [22]. A quaternion is defined as the following:

(1) $$q=q_{0}+q_{1}i+q_{2}j+q_{3}k$$

where the group of quaternions as defined by Hamilton in 1843 [23] utilizes the imaginary units that follow the definition $i^{2}=j^{2}=k^{2}=ijk=-1$ and $\left\{q_{0},q_{1},q_{2},q_{3}\in R\right\}$. It is also common to represent the quaternion as two components, the vector component (i,j, and k) and the scalar component (denoted by $q_{0}$). The purpose of the scalar component is to provide an additional, redundant parameter that keeps the quaternion fully defined in the event that a singularity may occur. This keeps the quaternion singularity free. Another way of thinking of it is thus:

(2) $$q=(q_{0},\bar{q})$$

Note that $q_{0}\in R$ and $\bar{q}={\left[q_{1},q_{2}.q_{3}\right]}^{T}\in R^{3}$. Additionally, the quaternion may sometimes be defined with the scalar component last as $q_{4}$. However, for the purposes of this paper, the quaternion always uses the scalar component as the first element. In practice, the scalar component tells the angle of rotation, and the normalized vector component provides the direction of the rotation axis.

The application of the quaternion itself is one that defines the rotation transformation of a coordinate frame. It is constructed using the following equation:

(3) $$q=\left(\cos\left(\frac{\theta }{2}\right),\bar{n}\sin\left(\frac{\theta }{2}\right)\right)$$

with $\bar{n}$ being the unit Euler axis [i,j,k] and θ being the angle of rotation in radians, both in three-dimensional space. Since rotations can be described by three parameters, the unit norm constraint is imposed on the quaternion for attitude representation. This norm constraint is an additional equation that fully constrains the quaternion in the event of an angular singularity. A quaternion describing the orientation of the X frame to the Y frame $\left(q_{X/Y}\right)$ satisfies the condition ${\left(q_{X/Y}\right)}^{*}\left(q_{X/Y}\right)=\left(q_{X/Y}\right){\left(q_{X/Y}\right)}^{*}=1$, where $1≜(1,{\bar{0}}_{3\times 1})$. There is a set of operations that handle the quaternion arithmetic. More details can be found in [24].

Three-dimensional vectors can also be interpreted as special cases of quaternions. This allows for the use of the quaternion along with vectors in three-dimensional space for equations that govern a rigid body dynamics. Redefining a vector ${\bar{s}}^{\times }$ such that ${\bar{s}}^{X}\in R^{3}$ in the frame of X to be in quaternion form is shown below:

(4) $$s^{X}=\left(s_{0},{\bar{s}}^{X}\right)\;\;\;\;\mathrm{with}\;\;s_{0}=0$$

Furthermore, the quaternion has explicit applications for changing reference frames. The change of reference frame on a vector in quaternion form to the Y frame from the X frame is achieved by the following:

(5) $$s^{Y}=q_{Y/X}^{*}s^{X}q_{Y/X}$$

Following this method replaces using a direction cosine matrix for frame transformation and is essential to the dynamics to follow.

### 2.2. Dual Numbers

Similar to how complex numbers consist of two parts known as real and complex, dual numbers are broken up into real and dual [25].

(6) $$z=a+\epsilon b\;\;\;\;\mathrm{where}\;\epsilon^{2}=0,\;\;\;\;\mathrm{but}\;\epsilon \neq 0$$

With this notation, *a* is the real number, and *b* is the dual number using ϵ as the distinguishing factor. With this concept in place, the dual number theory can be extended to other concepts such as vectors, real numbers, or even quaternions.

### 2.3. Dual-Quaternion

The dual-quaternion, denoted by a bold symbol, is comprised of eight components. This can be assigned such that a dual-quaternion has a real component and a dual component, where both the real and dual components are quaternions. As such, both the real and dual-quaternion components have their own scalar and vector components, respectively.

(7) $$q=q_{r}+\epsilon q_{d}$$

Now, as for the DQ mathematical operations, they generally follow the same guidelines as that of the quaternion. However, the added dual component requires that special attention be brought to grouping of the terms (real and dual), as well as the fact that the definition of the dual number is such that two dual numbers multiplied by each other equate to zero. There is a set of operations that handle the dual-quaternion arithmetic. More details can be found in [26]. Table 1 lists a simplified version of dual-quaternion operations and also lists the quaternion operations for convenience that are used in tandem with DQ operations.

The benefit of the dual-quaternion is the ability to group the equations that govern rotational motion and translational motion into a single equation. This is done by (typically) allotting the rotational components as the real quaternion component and using the dual-quaternion component for the translation.

While defining operators, it is worth pointing out the ⋆ operator [26]. Functionally, it performs identically to the traditional matrix multiplier. However, it is a special case as it has a specific definition for multiplying an 8 × 8 matrix with a dual-quaternion, the details of which will not be discussed in depth, as substituting it for classic matrix multiplication works just the same.

## 3. System Dynamics: Proximity Operation

**Figure 1:**
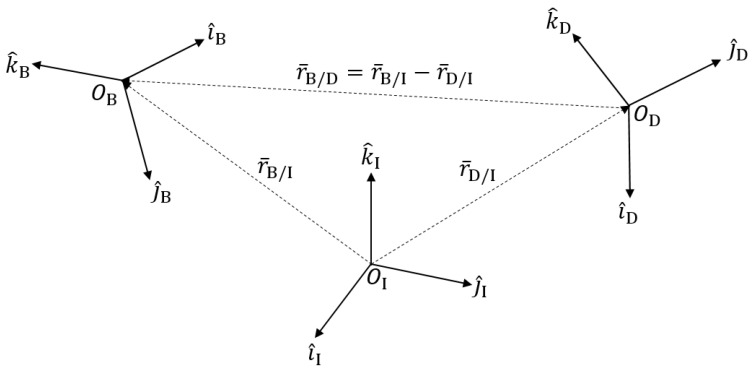
Relation between relative coordinate frames.

The previous dynamics were focused on utilizing the dual-quaternion for the two-body problem of a body in space while it orbits a planet—Earth, specifically. From here on, the proximity operation, or relative error dynamics, are discussed and built back to the dual-quaternion. With this, there is a desired trajectory, orientation, rotation rates, etc., from which a body is measured relatively and can be controlled to the desired frame. Figure 1 depicts both the body B frame and desired D frame relative to the inertial I frame to calculate the relative error. Then, using vector subtraction, the relativity of the body B frame from the desired D frame (denoted B/D) was calculated for control use.

### 3.1. Quaternion Dynamics

Upon converting all three-dimensional vectors into quaternion format using Equation (4), both the dynamics and kinematics require the use of the quaternion operations found in Table 1, after which, the translational and rotational equations of motion calculated for the body frame relative to the desired frame are [27]:

(8) $${\dot{r}}_{B/D}^{B}=v_{B/D}^{B}+\omega_{B/D}^{B}\times r_{B/D}^{B}$$

(9) $$\begin{array}{cc} m{\dot{v}}_{B/D}^{B}= & f^{B}-m\left({\dot{v}}_{D/I}^{B}+\omega_{D/I}^{B}\times r_{B/D}^{B}\right)-m\omega_{B/D}^{B}\times v_{B/D}^{B}\\ \; & -2m\omega_{D/I}^{B}\times v_{B/D}^{B}-m\omega_{D/I}^{B}\times v_{D/I}^{B}-m\omega_{D/I}^{B}\times \left(\omega_{D/I}^{B}\times r_{B/D}^{B}\right) \end{array}$$

(10) $${\dot{q}}_{B/D}=\frac{1}{2}q_{B/D}^{B}\omega_{B/D}^{B}$$

(11) $$\begin{array}{cc} I^{B}{\dot{\omega }}_{B/D}^{B}= & \tau^{B}-\left(\omega_{B/D}^{B}+\omega_{D/I}^{B}\right)\times \left(\left[I^{B}\right]\left(\omega_{B/D}^{B}+\omega_{D/I}^{B}\right)\right)\\ \; & -\left[I^{B}\right]\left(q_{B/D}^{*}{\dot{\omega }}_{D/I}^{D}q_{B/D}\right)-\left[I^{B}\right]\left(\omega_{D/I}^{B}\times \omega_{B/D}^{B}\right) \end{array}$$

where $f^{B}=\left(0,{\bar{f}}^{B}\right)$ are the body forces and $\tau^{B}=\left(0,{\bar{\tau }}^{B}\right)$ are the body torques. Equations (8) and (9) are the translational equations of motion, and Equations (10) and (11) are the rotational equations of motion of a frame fixed to a rigid body B relative to the desired reference frame D, both of which are propagating in the B frame.

The quaternion parameters within these equations consist of radius $r^{B}=\left(0,{\bar{r}}^{B}\right)$, velocity $v^{B}=\left(0,{\bar{v}}^{B}\right)$, angular velocity $\omega^{B}=\left(0,{\bar{\omega }}^{B}\right)$, and quaternion *q*. Exceptions include mass, *m*, which remains a scalar quantity, quaternion D/I variables, and $I^{B}$, which is defined below. This allows for the inertia matrix to be converted to a 4 × 4, so that it can be multiplied by the 4 × 1 of the quaternions.

(12) $$I^{B}=\left[\begin{array}{cc} 1 & 0_{1\times 3}\\ 0_{3\times 1} & {\bar{I}}^{B} \end{array}\right]$$

Note that ${\bar{I}}^{B}$ is still the 3×3 inertial matrix, but the bar is just to distinguish it from its 4×4 form for quaternion operations.

### 3.2. Dual-Quaternion Dynamics

Equations and variables in DQ form are both created by (typically) allocating the rotational quaternion components to be the real component of the dual-quaternion and having the translational components of motion be the dual component. The dual velocity (13) of a rigid body assigned to the B-frame from the D-frame, expressed in the body frame, is defined as:

(13) $$\omega_{B/D}^{B}=\omega_{B/D}^{B}+\epsilon \left(v_{B/D}^{B}\right)$$

Equation (13) contains the angular velocity as the real component, as well as the linear velocity as the dual component. Similarly, the dual-quaternion (quaternion parameter in dual form, not to be confused with other parameters in “DQ” form) is shown below.

(14) $$q_{B/D}=q_{B/D}+\epsilon \left(\frac{1}{2}q_{B/D}r_{B/D}^{B}\right)$$

It contains the unit quaternion $q_{B/D}$ as the real component for angular displacement and embeds $r^{B}$ within the dual term. It represents the attitude change from reference frame B to D, as well as the displacement.

The pose error kinematic equation of motion between frames B and D for the dual-quaternion is given by the following [28]:

(15) $${\dot{q}}_{B/D}=\frac{1}{2}q_{B/D}\omega_{B/D}^{B}$$

which accounts for the angular rate of change, as well as the rate of change of displacement. In the same method of combining the translational and rotational equations of motion (8) and (10) into dual-quaternions, the relative pose equation in DQ form [28] is created and embeds both Equations (9) and (11). With one equation of computation, both dynamic equations are accounted for.

(16) $$\begin{array}{cc} M^{B}⋆{\left({\dot{\omega }}_{B/D}^{B}\right)}^{S}= & f^{B}-\left(\omega_{B/D}^{B}+\omega_{D/I}^{B}\right)\times \left(M^{B}⋆\left({\left(\omega_{B/D}^{B}\right)}^{S}+{\left(\omega_{D/I}^{B}\right)}^{S}\right)\right)\\ \; & -M^{B}⋆{\left({\dot{\omega }}_{D/I}^{B}\right)}^{S}-M^{B}⋆{\left(\omega_{D/I}^{B}\times \omega_{B/D}^{B}\right)}^{S} \end{array}$$

where $f^{B}=f^{B}+\epsilon \tau^{B}$ is the dual-force applied on a body about its center of mass and $M^{B}$ creates an 8×8 diagonal matrix containing both the mass and the moment of inertia in order to pair with the 8×1 quaternion array.

(17) $$M^{B}≜\left[\begin{array}{cccc} 1 & 0_{1\times 3} & 0 & 0_{1\times 3}\\ 0_{3\times 1} & mI_{3} & 0_{3\times 1} & 0_{3\times 3}\\ 0 & 0_{1\times 3} & 1 & 0_{1\times 3}\\ 0_{3\times 1} & 0_{3\times 3} & 0_{3\times 1} & {\bar{I}}^{B} \end{array}\right]$$

where $I_{3}$ is a 3×3 identity matrix and ${\bar{I}}^{B}$ is still the 3×3 inertial matrix.

It is also important to take note of the general rule $\omega_{Y/Z}^{X}=q_{X/Y}^{*}\omega_{Y/Z}^{Y}q_{X/Y}=\omega_{Y/Z}^{X}+\epsilon \left(v_{Y/Z}^{X}+\omega_{Y/Z}^{X}\times r_{X/Y}^{X}\right)$ when constructing the dual-velocity variables that are not in the B-coordinate frame; one of specific interest would be $\omega_{D/I}^{B}=\omega_{D/I}^{B}+\epsilon \left(v_{D/I}^{B}+\omega_{D/I}^{B}\times r_{B/D}^{B}\right)$, as is the case with the identity ${\dot{\omega }}_{D/I}^{B}=\left(q_{B/D}^{*}{\dot{\omega }}_{D/I}^{D}q_{B/D}\right)$.

### 3.3. External Forces and Dynamic Disturbances

The typical decomposition of $f^{B}$ includes the total external dual-force acting on an Earth-orbiting spacecraft, including control and all specified disturbance.

(18) $$\begin{array}{c} f^{B}=f_{g}^{B}+f_{J_{2}}^{B}+f_{\nabla g}^{B}+f_{c}^{B} \end{array}$$

where $f_{g}^{B}$ is the two-body dual-gravitational force, $f_{J_{2}}^{B}$ is the dual-force due to spherical harmonic perturbations, $f_{\nabla g}^{B}$ is the dual-gravity gradient force, and $f_{c}^{B}$ is the dual-control force.

Calculating the vector forces and vector torques is necessary prior to conversion to DQ format. $f_{g}^{B}$ is the dual-gravitational force where $f_{g}^{B}$$=f_{g}^{B}+\epsilon 0$, where $f_{g}^{B}=\left(0,m{\bar{a}}_{g}^{B}\right)$. Similarly, $f_{\nabla g}^{B}=0+\epsilon \tau_{\nabla g}^{B}$ where $\tau_{\nabla g}^{B}=\left(0,{\bar{\tau }}_{\nabla g}^{B}\right)$ and $f_{J_{2}}^{B}=f_{J_{2}}^{B}+\epsilon 0$ in that $f_{J_{2}}^{B}=\left(0,{\bar{a}}_{J_{2}}\right)$. The vector accelerations/torques are such that they all exist in $R^{3}$ in the Cartesian coordinate system and are as follows:

(19) $${\bar{a}}_{g}^{B}=-\frac{\mu {\bar{r}}_{B/I}^{B}}{||{\bar{r}}_{B/I}^{B}{\left|\right|}^{3}}$$

(20) $${\bar{\tau }}_{\nabla g}^{B}=3\mu \frac{{\bar{r}}_{B/I}^{B}\times \left(\bar{I}{\bar{r}}_{B/I}^{B}\right)}{\left|\right|{\bar{r}}_{B/I}^{B}{\left|\right|}^{5}}$$

(21) $${\bar{a}}_{J_{2}}=-\frac{3\mu J_{2}R_{e}^{2}}{2||{\bar{r}}_{B/I}^{I}{\left|\right|}^{5}}\left[\begin{array}{c} \left(1-5{\left(\frac{z_{B/I}^{I}}{\left|\right|r_{B/I}^{I}\left|\right|}\right)}^{2}\right)x_{B/I}^{I}\\ \left(1-5{\left(\frac{z_{B/I}^{I}}{\left|\right|{\bar{r}}_{B/I}^{I}\left|\right|}\right)}^{2}\right)y_{B/I}^{I}\\ \left(3-5{\left(\frac{z_{B/I}^{I}}{\left|\right|{\bar{r}}_{B/I}^{I}\left|\right|}\right)}^{2}\right)z_{B/I}^{I} \end{array}\right]$$

Additional body forces and torques may be incorporated, including atmospheric drag, solar drag, or additional celestial two-body forces. For simplification, these disturbances were not included, but could be added as necessary. In the simple undisturbed case, only $f_{g}^{B}$ and $f_{c}^{B}$ remain with all other terms taken as zero.

## 4. Control Design

### 4.1. Overview

**Figure 2:**
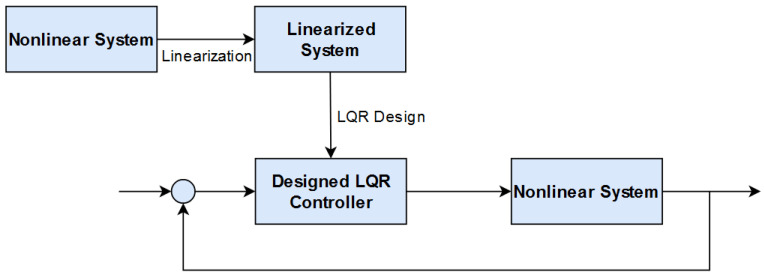
LQR controller design based on the linearized spacecraft model. LQR is the sub-optimal control and globally stabilizes the nonlinear spacecraft system.

Figure 2 illustrates the adopted control design procedure. Beginning with the nonlinear system, the equations of motion are linearized and produce a linearized system model. The process then results in the linear-quadratic regulator (LQR) controller design based on the linearized spacecraft model. Through LQR design, an optimal solution was achieved. Then, the optimal gains of the linearized system were plugged back into the original nonlinear system to produce a stable “sub-optimal” control, as well as to prove global stability for the nonlinear system.

With the nonlinear system discussed in detail in Section 4, the linearization process and LQR design are sequentially discussed.

### 4.2. Linearized System

The linearized dynamics of Equations (16) and (15) can be written in the general state form as follows:

(22) $$\dot{x}=Ax+Bu$$

where x is the state variable, u is the control input, *A* is the state matrix, and *B* is the control matrix. The x state variable for this controller incorporates the necessary states that instantaneously define a rigid body displacement, orientation, velocity, and rotational velocity relative to the body frame B. It is defined using the four quaternion terms:

(23) $$x=\left\{\begin{array}{c} \omega_{B/D}^{B}\\ v_{B/D}^{B}\\ q_{B/D}\\ \frac{1}{2}q_{B/D}r_{B/D}^{B} \end{array}\right\}$$

or, putting things into the preferred dual-quaternion format:

(24) $$x=\left\{\begin{array}{c} \omega_{B/D}^{B}\\ q_{B/D} \end{array}\right\}$$

where the state variable x is a 16×1 state vector consisting of its two dual-quaternion elements. This then leads to:

(25) $$\dot{x}=\left\{\begin{array}{c} {\dot{\omega }}_{B/D}^{B}\\ {\dot{q}}_{B/D} \end{array}\right\}$$

Returning to Equation (22), the *A* and *B* matrices are 16×16 and 16×8 matrices, respectively. The linearized state matrix and linear control matrix were constructed by performing the Jacobian operation on $\dot{x}$ with respect to the state variable x and then performing the arbitrary substitution $x=0≜{\left[0,0,0,0,0,0,0,0,1,0,0,0,0,0,0,0\right]}^{T}$ (note the exception of $q_{B/D}={\left[1,0,0,0\right]}^{T}$) to obtain linearity such that $\frac{\partial \dot{x}}{\partial x}{|}_{x=0}$. The dynamics were assumed to stabilize around x=0; i.e., zero steady-state error in the relative motion. Linearization was performed about this equilibrium point. Recalling the dual-quaternion Equations (15) and (16) to obtain $\dot{x}$ in Equation (25), the state matrices were constructed as:

(26) $$A=\left[\begin{array}{cc} \frac{\partial {\dot{\omega }}_{B/D}^{B}}{\partial \omega_{B/D}^{B}}\; & \frac{\partial {\dot{\omega }}_{B/D}^{B}}{\partial q_{B/D}}\\ \frac{\partial {\dot{q}}_{B/D}}{\partial \omega_{B/D}^{B}}\; & \frac{\partial {\dot{q}}_{B/D}}{\partial q_{B/D}} \end{array}\right]\;\;\;\;\;$$

(27) $$B=\left[\begin{array}{c} \frac{\partial {\dot{\omega }}_{B/D}^{B}}{\partial f^{B}}\\ \frac{\partial {\dot{q}}_{B/D}}{\partial f^{B}} \end{array}\right]$$

For the ease of understanding, the *A* matrix is presented in ordinary quaternion format below. Due to the last quaternion of the state variable being the dual component $\frac{1}{2}q_{B/D}r_{B/D}^{B}$ of $q_{B/D}$, that is $q_{B/D}=q_{B/D}+\epsilon \left(\frac{1}{2}q_{B/D}r_{B/D}^{B}\right)$, the chain rule needs to be taken into consideration with regard to these partial derivatives. As such, a dummy variable $\alpha_{B/D}^{B}$ such that $\alpha_{B/D}^{B}=\frac{1}{2}q_{B/D}r_{B/D}^{B}$ was utilized for the ease of presentation. Using the chain rule, this led to ${\dot{\alpha }}_{B/D}^{B}=\frac{1}{2}q_{B/D}{\dot{r}}_{B/D}^{B}+\frac{1}{4}r_{B/D}^{B}q_{B/D}\omega_{B/D}^{B}$.

(28) $$A=\left[\begin{array}{cccc} \frac{\partial {\dot{\omega }}_{B/D}^{B}}{\partial \omega_{B/D}^{B}}\; & \frac{\partial {\dot{\omega }}_{B/D}^{B}}{\partial v_{B/D}^{B}}\; & \frac{\partial {\dot{\omega }}_{B/D}^{B}}{\partial q_{B/D}}\; & \frac{\partial {\dot{\omega }}_{B/D}^{B}}{\partial \alpha_{B/D}^{B}}\\ \frac{\partial {\dot{v}}_{B/D}^{B}}{\partial \omega_{B/D}^{B}}\; & \frac{\partial {\dot{v}}_{B/D}^{B}}{\partial v_{B/D}^{B}}\; & \frac{\partial {\dot{v}}_{B/D}^{B}}{\partial q_{B/D}}\; & \frac{\partial {\dot{v}}_{B/D}^{B}}{\partial \alpha_{B/D}^{B}}\\ \frac{\partial {\dot{q}}_{B/D}}{\partial \omega_{B/D}^{B}}\; & \frac{\partial {\dot{q}}_{B/D}}{\partial v_{B/D}^{B}}\; & \frac{\partial {\dot{q}}_{B/D}}{\partial q_{B/D}}\; & \frac{\partial {\dot{q}}_{B/D}}{\partial \alpha_{B/D}^{B}}\\ \frac{\partial {\dot{\alpha }}_{B/D}^{B}}{\partial \omega_{B/D}^{B}}\; & \frac{\partial {\dot{\alpha }}_{B/D}^{B}}{\partial v_{B/D}^{B}}\; & \frac{\partial {\dot{\alpha }}_{B/D}^{B}}{\partial q_{B/D}}\; & \frac{\partial {\dot{\alpha }}_{B/D}^{B}}{\partial \alpha_{B/D}^{B}} \end{array}\right]\;\;\;\;\;$$

Finally, the two matrices were evaluated, and the substitution x=0 was performed. On the following page, the linearized *A* matrix is given in Equation (30), and the linearized *B* matrix shown in Equation (29) is also in a compact form, with mass *m* and $I^{B}$ as the 3×3 inertia matrix in the B frame.

(29) $$B=\left[\begin{array}{cccc} 0 & 0_{1\times 3} & 0 & 0_{1\times 3}\\ 0_{3\times 1} & {I^{B}}^{-1} & 0_{3\times 1} & 0_{3\times 3}\\ 0_{3\times 1} & 0_{3\times 3} & 0_{3\times 1} & m^{-1}I_{3}\\ 0_{8\times 1} & 0_{8\times 3} & 0_{8\times 1} & 0_{8\times 3} \end{array}\right]$$

(30) $$\;A=\left[\begin{array}{ccccccccc} 0 & 0 & 0 & 0 & 0 & 0 & 0 & 0 & 0_{1\times 8}\\ 0 & 0 & \frac{(\omega_{3\;D/I}\left(I_{1}+I_{2}-I_{3}\right)}{I_{1}} & \frac{-(\omega_{2\;D/I}\left(I_{1}-I_{2}+I_{3}\right)}{I_{1}} & 0 & 0 & 0 & 0 & 0_{1\times 8}\\ 0 & \frac{-(\omega_{3\;D/I}\left(I_{1}+I_{2}-I_{3}\right)}{I_{2}} & 0 & \frac{(\omega_{1\;D/I}\left(I_{2}-I_{1}+I_{3}\right)}{I_{2}} & 0 & 0 & 0 & 0 & 0_{1\times 8}\\ 0 & \frac{(\omega_{2\;D/I}\left(I_{1}-I_{2}+I_{3}\right)}{I_{3}} & \frac{-(\omega_{1\;D/I}\left(I_{2}-I_{1}+I_{3}\right)}{I_{3}} & 0 & 0 & 0 & 0 & 0 & 0_{1\times 8}\\ 0 & 0 & 0 & 0 & 0 & 0 & 0 & 0 & 0_{1\times 8}\\ 0 & 0 & 0 & 0 & 0 & 0 & 2\omega_{3\;D/I} & {-2\omega }_{2\;D/I} & 0_{1\times 8}\\ 0 & 0 & 0 & 0 & 0 & -2\omega_{3\;D/I} & 0 & {-2\omega }_{1\;D/I} & 0_{1\times 8}\\ 0 & 0 & 0 & 0 & 0 & 2\omega_{2\;D/I} & {-2\omega }_{1\;D/I} & 0 & 0_{1\times 8}\\ 0 & 1/2 & 0 & 0 & 0 & 0 & 0 & 0 & 0_{1\times 8}\\ 0 & 0 & 1/2 & 0 & 0 & 0 & 0 & 0 & 0_{1\times 8}\\ 0 & 0 & 0 & 1/2 & 0 & 0 & 0 & 0 & 0_{1\times 8}\\ 0 & 0 & 0 & 0 & 0 & 0 & 0 & 0 & 0_{1\times 8}\\ 0 & 0 & 0 & 0 & 0 & 1/2 & 0 & 0 & 0_{1\times 8}\\ 0 & 0 & 0 & 0 & 0 & 0 & 1/2 & 0 & 0_{1\times 8}\\ 0 & 0 & 0 & 0 & 0 & 0 & 0 & 1/2 & 0_{1\times 8} \end{array}\right]$$

As a side note, through inspection, the linearized equations can be obtained in an alternate form through substitution of *A* and *B* into Equation (22). First, let us fully expand (16) and perform a sanity check so what we may look for in the linear terms, as well as isolate the nonlinear terms.

(31) $$\begin{array}{cc} \;{\left({\dot{\omega }}_{B/D}^{B}\right)}^{S}= & -\underset{\mathrm{linear}\;\mathrm{terms}}{\underset{︸}{{\left(M^{B}\right)}^{{}^{-1}}\;⋆\left(\left(\omega_{B/D}^{B}\;\times \;M^{B}\;⋆{\left(\omega_{D/I}^{B}\right)}^{S}\right)+\left(\omega_{D/I}^{B}\;\times \;M^{B}\;⋆{\left(\omega_{B/D}^{B}\right)}^{S}\right)\right)-{\left(\omega_{D/I}^{B}\;\times \;\omega_{B/D}^{B}\right)}^{S}}}\\ \; & \underset{\mathrm{nonlinear}\;\mathrm{terms}}{\underset{︸}{-{\left(M^{B}\right)}^{{}^{-1}}\;⋆\left(\omega_{B/D}^{B}\times M^{B}⋆{\left(\omega_{B/D}^{B}\right)}^{S}\right)}}\\ \; & \underset{\mathrm{function}\;\mathrm{of}\;\mathrm{time}\;\mathrm{terms}}{\underset{︸}{-{\left(M^{B}\right)}^{{}^{-1}}\;⋆\left(\omega_{D/I}^{B}\times M^{B}⋆{\left(\omega_{D/I}^{B}\right)}^{S}\right)-{\left({\dot{\omega }}_{D/I}^{B}\right)}^{S}}}\underset{\mathrm{control}}{\underset{︸}{+{\left(M^{B}\right)}^{{}^{-1}}\;⋆f^{B}}} \end{array}$$

The creation of (31) shows the separation of Equation (16) such that it is comprised of the linear terms, the nonlinear terms, the control, and the terms labeled “Other”. These other terms are not necessarily nonlinear, but they do not appear in the linearized equation due to it being linearized around the selected state variable of $\omega_{B/D}^{B}$. Once linearized, the equation becomes the following:

(32) $$\begin{array}{cc} {\left({\dot{\omega }}_{B/D}^{B}\right)}^{S}=-{\left(M^{B}\right)}^{{}^{-1}}⋆\;\left(\left(\omega_{B/D}^{B}\times M^{B}⋆{\left(\omega_{D/I}^{B}\right)}^{S}\right)+\left(\omega_{D/I}^{B}\times M^{B}⋆{\left(\omega_{B/D}^{B}\right)}^{S}\right)\right)\; & \\ -{\left(\omega_{D/I}^{B}\times \omega_{B/D}^{B}\right)}^{S}+{\left(M^{B}\right)}^{{}^{-1}}⋆f^{B} \end{array}$$

Likewise, the linearization of (15), ${\dot{q}}_{B/D}$ may be found below. Both Equations (32) and (33) match up with the linear dynamics as described by (22) using the *A* and *B* matrices and were derived by inspection using these. These linearized equations may serve as a substitute for the linear dynamics, but for the purposes of this manuscript, they are only informational and were not used in place of the A and B matrices in conjunction with (22).

(33) $${\dot{q}}_{B/D}=\frac{1}{2}\omega_{B/D}^{B}$$

### 4.3. Optimal LQR Design of the Linearized System

The LQR design was obtained from the “controllable” linearized equation by minimizing the quadratic performance index:

(34) $$J=\frac{1}{2}\int \left(x^{T}Qx+u^{T}Ru\right)dt$$

subject to the state equation, Equation (22), with the newly found *A* and *B* matrices. *Q* and *R* are weighting matrices. The control solution for this problem is the linear state feedback control $u=-R^{-1}B^{T}Sx$, where *S* is the state feedback gain that is calculated by solving the Riccati equation:

(35) $$A^{T}S+SA-SBR^{-1}B^{T}S+Q=0$$

An optimal control problem was designed by minimizing the performance index given in Equation (34) subject to the state Equation (22). The input *u* in (22) was a contribution of the optimal control force and the body dynamic force, as seen in (18). Thus, Equation (22) can be written as follows:

(36) $$\dot{x}=Ax+Bu^{*}+d$$

where $u^{*}=f_{c}$ is the optimal control and *d* consists of all the other “function of time” terms and other body forces:

(37) $$\begin{array}{c} d=f_{g}^{B}+f_{J_{2}}^{B}+f_{\nabla g}^{B} \end{array}$$

Invoking the necessary optimization condition and including disturbance rejection terms in the feedback gain, the optimal linear state feedback control yields:

(38) $$u^{*}=-R^{-1}B^{T}Sx-R^{-1}B^{T}v$$

where *S* is the state feedback gain found by solving the Riccati Equation (35) and *v* is the disturbance rejection gain [29,30]:

(39) $$v={\left[{\left(A-BR^{-1}B^{T}S\right)}^{T}\right]}^{-1}Sd$$

The linearized spacecraft system is fully controllable using this LQR design.

### 4.4. Sub-Optimal Nonlinear Feedback Control Design

This section treats the nonlinear dynamics in tensor notation. Therefore, the performance index in Equation (34) can be written as in Equation (40). A finite-time optimal control problem was designed by minimizing the following universal performance index:

(40) $$\begin{array}{cc} J= & \frac{1}{2}\Phi \left(t_{f},x\left(t_{f}\right)\right)+\frac{1}{2}\int_{t_{0}}^{t_{f}}\left\{x^{T}Qx+u^{T}Ru\right\}\;dt\\ \;= & \frac{1}{2}\Phi \left(t_{f},x_{i}\left(t_{f}\right)\right)+\frac{1}{2}\int_{t_{0}}^{t_{f}}\left\{q_{j_{3}j_{4}}x_{j_{3}}x_{j_{4}}+r_{j_{5}j_{6}}u_{j_{5}}u_{j_{6}}\right\}\;dt \end{array}$$

subject to the nonlinear state Equation (31), given in polynomial tensor form:

(41) $${\dot{x}}_{i}=a_{ij_{1}}x_{j_{1}}+c_{ijkl}x_{j}x_{k}x_{l}+t_{imn}x_{m}x_{n}+d_{i}+b_{ij_{2}}u_{j_{2}}$$

where $x=x_{i}$ contains the state variables, $t_{0}$ is the initial time, and $t_{f}$ is the final time. Note that the state coefficients depend on the system dynamics. For instance, $a_{ij_{1}}$ is the coefficient of the linear term, while $t_{imn}$ and $c_{ijkl}$ are the coefficient tensors of the nonlinear terms. The dynamic disturbance is represented by the vector $d_{i}$. $\Phi (t_{f},x_{i}\left(t_{f}\right))$ is a soft terminal constraint that is a function of the final time $t_{f}$ and the final state $x_{f}$. Tensor indices depend on the number of dynamics states. Invoking the necessary optimization conditions yields the following control law:

(42) $$u_{j_{6}}=-r_{i_{3}j_{6}}^{-1}b_{i_{2}i_{3}}\lambda_{i_{2}}$$

where $r_{i_{3}j_{6}}^{-1}$ denotes the $i_{3}j_{6}$-th element in the inverse of matrix R (with elements defined by $r_{i_{3}j_{6}}$) and $\lambda_{i_{2}}$ is the co-state variable. A tensor-based co-state structure was assumed, which led to tensor-based gain equations that define the time history for the feedback control solution. Following the standard linear feedback control strategy, a generalized nonlinear feedback co-state structure was assumed to account for the quadratic nonlinearity in the dynamics.

(43) $$\lambda_{i_{4}}=s_{i_{4}j_{9}j_{10}}x_{j_{9}}x_{j_{10}}+k_{i_{4}j_{8}}x_{j_{8}}+p_{i_{4}}$$

where $s_{i_{4}j_{9}j_{10}}$, $k_{i_{4}j_{8}}$, and $p_{i_{4}}$ are the control gains sought. Thus, the control input is given by:

(44) $$\begin{array}{c} u_{j_{6}}=-r_{i_{5}j_{6}}^{-1}b_{i_{6}i_{5}}s_{i_{6}j_{31}j_{32}}x_{j_{31}}x_{j_{32}}-r_{i_{5}j_{6}}^{-1}b_{i_{6}i_{5}}k_{i_{6}j_{30}}x_{j_{30}}-r_{i_{5}j_{6}}^{-1}b_{i_{6}i_{5}}p_{i_{6}} \end{array}$$

Therefore, the gain differential equations can be written as:

(45) $$\begin{array}{cc} {\dot{p}}_{i}= & -k_{ij}d_{j}+k_{ij}b_{jk}r_{lk}^{-1}b_{ml}p_{m}-p_{n}a_{ni} \end{array}$$

(46) $$\begin{array}{cc} {\dot{k}}_{ij}= & -k_{kj}a_{ki}+k_{il}b_{lm}r_{nm}^{-1}b_{on}k_{oj}-k_{il}a_{lj}-s_{ijp}d_{p}-s_{iqj}d_{q}\\ \; & +s_{iqj}b_{qm}r_{nm}^{-1}b_{on}p_{o}+s_{ijp}b_{pm}r_{nm}^{-1}b_{on}p_{o}-q_{ij}-p_{r}t_{rji}-p_{r}t_{rij} \end{array}$$

(47) $$\begin{array}{cc} {\dot{s}}_{ijk}= & -k_{lk}t_{lji}-k_{lk}t_{lij}-s_{ljk}a_{li}-k_{im}t_{mjk}-s_{inj}a_{nk}\\ \; & +k_{im}b_{mo}r_{po}^{-1}b_{qp}s_{qjk}+s_{ink}b_{no}r_{po}^{-1}b_{qp}k_{qj}-s_{ijr}a_{rk}\\ \; & +s_{ikr}b_{ro}r_{po}^{-1}b_{qp}k_{qj}-p_{s}c_{sjki}-p_{s}c_{sjik}-p_{s}c_{sijk} \end{array}$$

The gain differential equations are integrated backwards in time with the following boundary conditions: $p_{i}\left(t_{f}\right)=0$, $k_{ij}\left(t_{f}\right)=K_{f}$, and $s_{ijk}\left(t_{f}\right)=0$. The above generalized feedback dynamics provide a rigorous technique to handle system nonlinearity effects and disturbances. The tensor-based development of the 3 DOF attitude motion optimal control problem using the classical quadratic penalty function was detailed in [29,30]. The resulting gain equations obtained for this combined translational and rotational control were based on the selected 6 DOF dynamics built on the full 6 DOF nonlinear controller presented in this paper.

## 5. Simulation Results

This section presents the simulation results of the nonlinear dual-quaternion dynamics of the proximity operations with various control solutions. The nonlinear dynamics, linear dynamics, and suboptimal nonlinear dynamics for the dual-quaternion proximity operation equations using the methods described in this paper were simulated in MATLAB for the Earth orbit of two satellite bodies and propagated using the built-in MATLAB function ODE45. All dual-quaternion and quaternion functions used in the simulation were created for this paper using the presented equations and theory.

The initial state variable conditions for this example are given in Table 2. The data below serve as an aid to help understand the context of the simulation. The simulation variables were selected to provide a simple elliptical orbit around Earth to allow the dynamics a simulated testing environment. The masses of the satellites $m_{B}$ and $m_{D}$, as well as the intertias $I_{B}$ and $I_{D}$ were selected to represent a simple cube-sat. The final simulation time $t_{f}$, as well as variables in the B/D frame were selected to be relatively tight to show real-world applicability to a problem such as this.

### 5.1. Non-Linear Dual-Quaternion with Arbitrary Control Gains

**Figure 3:**
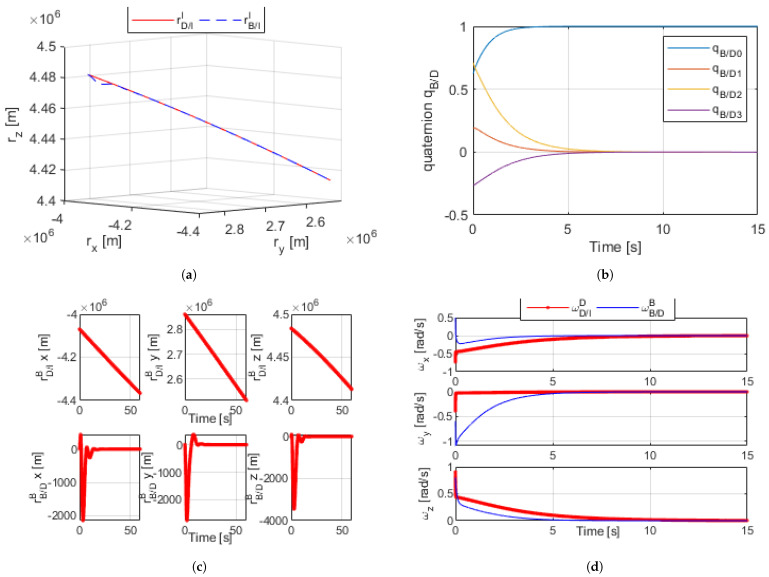
(**a**) Dual quaternion proximity orbital trajectory simulation [60 s]. (**b**) Dual-quaternion relative angles [15 s]. (**c**) Dual-quaternion proximity radius (desired and relative) [60 s]. (**d**) Dual-quaternion proximity angular velocity (desired and relative) [15 s].

Figure 3a–d shows the results for simulating the proximity operation with arbitrary control gains using the dual-quaternion. Upon using a set of arbitrary initial conditions that simulate an orbit of a body along a desired trajectory, as well as another pair of arbitrary initial conditions for $\omega_{B/D}^{B}$ and $q_{B/D}$ in DQ format, Equations (16) through (18) were used to continuously calculate the error and relatively control the body in the B frame to match that in the D frame while subject to disturbances.

Figure 3a shows an inertial I frame with the trajectory path of both bodies, as $r_{B/I}^{I}$ is controlled to match the desired trajectory $r_{D/I}^{I}$. Figure 3b plots $q_{B/D}$, the angular difference between bodies B and D, as the body in the B frame was controlled such that the difference between the two was zero. Figure 3c reflects how the relative position between the two bodies became zero, allowing $r_{B/I}^{I}$ to match $r_{D/I}^{I}$. Figure 3d shows $\omega_{B/D}^{B}$—the differences between the angular rates of B and D—reaching zero. The accumulation of these results proved the validity of the dual-quaternion for this method of control.

### 5.2. Optimal Linearized Dual-Quaternion

Building on the dual-quaternion for relative dynamics, the same simulation was run using the linearized proximity equation in the dual-quaternion. Putting the linearized state matrix *A* and control matrix *B* into the general state form of Equation (22) allowed for the control of the linearized state in DQ form. The same premise was in place; the error of the body in the B frame relative to that in the D frame was continuously calculated and controlled to zero while subject to disturbances. The same initial conditions were in place.

**Figure 4:**
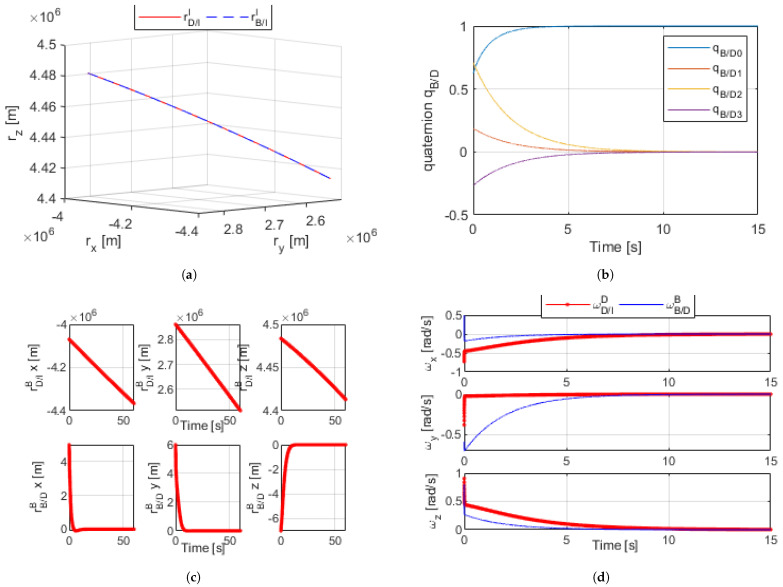
(**a**) Linear optimized dual-quaternion proximity simulation orbital trajectory [60 s]. (**b**) Linear optimized dual-quaternion relative angles [15 s]. (**c**) Linear optimized dual-quaternion proximity radius [60 s]. (**d**) Linear optimized dual-quaternion proximity angular velocity [15 s].

The results of the optimal dual-quaternion can be found in Figure 4a–d. All state variables achieved convergence well before the final time of the simulation, so the figures shown are cropped for emphasis; the relative error between B and D reached zero. The results of the optimal linearized model showed dramatic improvement from the nonlinear DQ proximity operation with arbitrary gains.

Table 3 shows the initial conditions used to run a Monte Carlo simulation for the linear dual-quaternion model. It shows the same initial conditions of the prior results, but with random values for all the variables of the B/D frame using r∈R such that −1≤r≤1 as a random independent number with no relation to any other use of this random number. The probability distribution associated with *r* inherently was uniform across the range of r. Note that even though random, $q_{B/D}$ was still normalized using $q_{B/D}=\frac{q_{B/D}}{||q_{B/D}||}$.

**Figure 5:**
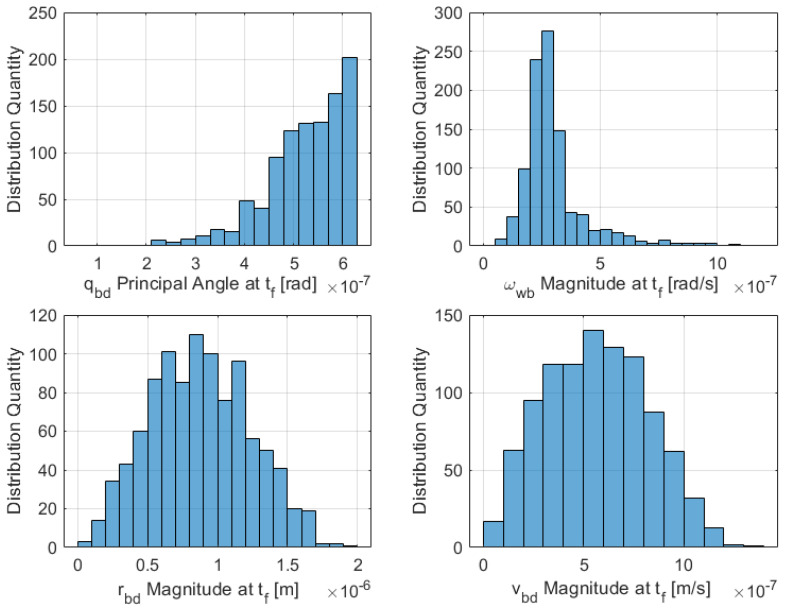
Monte Carlo random initial conditions histogram for linearized DQ proximity operation-magnitude of the state variables at the final time (1000 runs).

The results of a Monte Carlo simulation for the linearized dual-quaternion proximity operation can be seen in Figure 5. Below is a histogram of the final norm values for the relative angle, relative angular velocity, relative position, and relative velocity from B to D at final time $t_{f}$. The simulation utilized a data pool of 1000 runs with random initial conditions. Compiling all final values into a histogram plot shows the range and distribution of possible values to demonstrate convergence to near zero.

### 5.3. Non-Linear Near Optimal and Stable Dual-Quaternion

**Figure 6:**
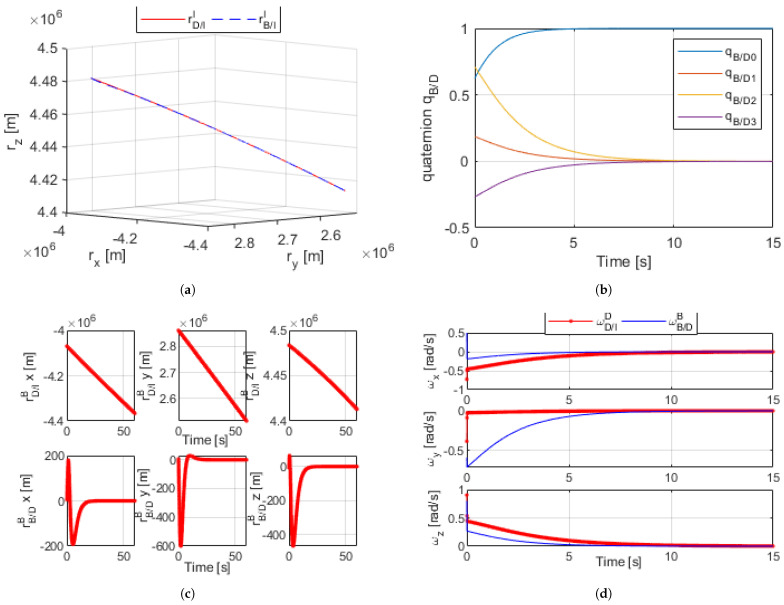
(**a**) Nonlinear sub-optimal dual-quaternion proximity orbital simulation [60 s]. (**b**) Nonlinear sub-optimal dual-quaternion angles [15 s]. (**c**) Nonlinear sub-optimal dual-quaternion proximity radius [60 s]. (**d**) Nonlinear sub-optimal dual-quaternion proximity angular velocity [15 s].

**Figure 7:**
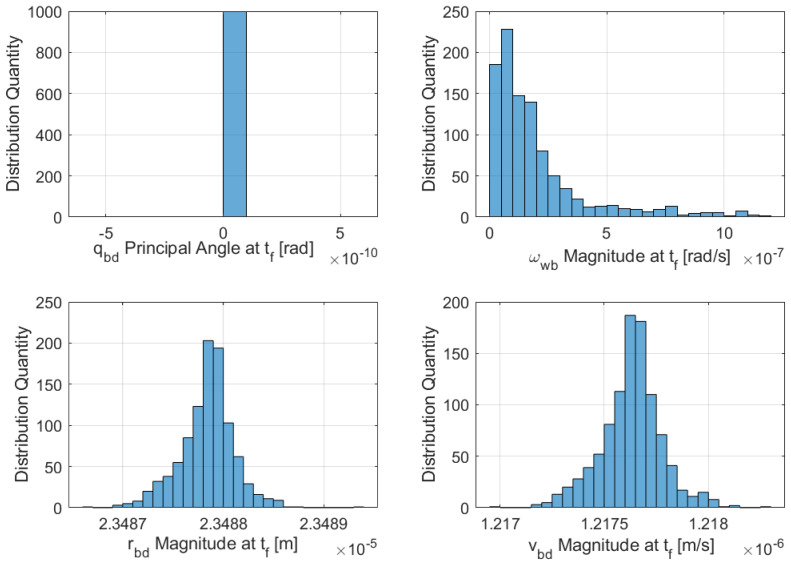
Monte Carlo random initial conditions histogram for sub-optimal DQ proximity operation—magnitude of the state variables at the final time (1000 runs).

The generalized higher order feedback control was applied to the full nonlinear DQ equations using the outlined suboptimal nonlinear feedback controller. The results are presented in Figure 6a–d, with global stability obtained well before the simulation’s final time by observing that all state variables achieved convergence; the relative error between the B and D frames reached zero. Figure 7 presents the results of another 1000 trial Monte Carlo runs with random gains showing final state variable magnitudes. Furthermore, sub-optimal optimization was achieved by considering the optimal feedback solution in the higher order gains.

The results between the linear optimal and sub-optimal experiments showed striking similarity for the rotational dynamics of $q_{B/D}$ and $\omega_{B/D}$. The most notable difference was the performance of the translational dynamics via the sub-optimal controller, which was vast improvement from the arbitrary non-linear controller. Otherwise, all dual-quaternion simulations achieved their desired final states. These results were also backed by the Monte Carlo simulations.

## 6. Conclusions

Using dual-quaternions, an alternative representation of the translational and rotational equations of motion for a rigid body were combined and utilized for applications in satellite dynamics. All necessary formulations based on quaternion equivalencies were also presented and verified when compared to their dual-quaternion pairing. Applications for using the dual-quaternion in satellite dynamics that were discussed within this paper included classical orbits and satellite proximity control. A linearized model was developed for the relative motion using Taylor’s expansion of the first order and stabilized about an equilibrium point to achieve linearized equations of motion in a dual-quaternion framework. The stability of the linearized model was based on LQR and verified with a Monte Carlo simulation. The sub-optimal control law using generalized higher order feedback dual-quaternion gains was developed using small error approximations for the proximity operation and also verified for stability using a Monte Carlo simulation.

## Figures and Tables

**Table 1:** Dual-quaternion and quaternion operations.

Operation	Dual-Quaternion Definition	Quaternion Definition
Addition	$a+b=\left(a_{r}+b_{r}\right)+\epsilon \left(a_{d}+b_{d}\right)$	$a+b=(a_{0}+b_{0},\bar{a}+\bar{b})$
Scalar multiplication	$\lambda a=\left(\lambda a_{r}\right)+\epsilon \left(\lambda a_{d}\right)$	$\lambda a=(\lambda a_{0},\lambda \bar{a})$
Multiplication	$\mathrm{ab}=\left(a_{r}b_{r}\right)+\epsilon \left(a_{d}b_{r}+a_{r}b_{d}\right)$	$ab=(a_{0}b_{0}-\bar{a}\;\cdot \;\bar{b},a_{0}\bar{b}+b_{0}\bar{a}+\bar{a}\times \bar{b})$
Conjugate	$a^{*}=\left(a_{r}^{*}\right)+\epsilon \left(a_{d}^{*}\right)$	$a^{*}=\left(a_{0},-\bar{a}\right)$
Dot product	$a\;\cdot \;b=\left(a_{r}\;\cdot \;b_{r}\right)+\epsilon \left(a_{d}\;\cdot \;b_{r}+a_{r}\;\cdot \;b_{d}\right)$	$a\;\cdot \;b=(a_{0}b_{0}+\bar{a}\;\cdot \;\bar{b},0_{3\times 1})$
Cross product	$a\times b=\left(a_{r}\times b_{r}\right)+\epsilon \left(a_{d}\times b_{r}+a_{r}\times b_{d}\right)$	$a\times b=(0,a_{0}\bar{b}+b_{0}\bar{a}+\bar{a}\times \bar{b}$)
Norm	$||a||=\sqrt{(a_{r}\;\cdot \;a_{r}+a_{d}\;\cdot \;a_{d})+\epsilon 0}$	$||a||=\sqrt{a\;\cdot \;a}$
Swap	$a^{S}=a_{d}+\epsilon a_{r}$	—

**Table 2:** Simulation initial condition inputs.

$t_{f}$	60 s
$m_{B}=m_{D}$	1.33 kg
$I_{B}=I_{D}$	$I_{3}$ [0.0017 0.0020 0.0022] kg m^2^
${\bar{r}}_{D/I}^{I}$	[−4,069,503 2,861,786 4,483,608] m
${\bar{v}}_{D/I}^{I}$	[−5114 −5691 −1000] m/s
${\bar{\omega }}_{D/I}^{D}$	[−1.0891 0.0326 0.5525] rad/s
$q_{D/I}$	[0.4559 0.6396 0.0356 −0.6179]
${\bar{r}}_{B/D}^{B}$	[5 6 −7] m
${\bar{v}}_{B/D}^{B}$	[0.5 −0.6 0.7] m/s
${\bar{\omega }}_{B/D}^{B}$	[0.5 −0.59 0.8] rad/s
$q_{B/D}$	[0.6230 0.1863 0.7110 −0.2678]

**Table 3:** Monte Carlo initial condition inputs.

$t_{f}$	60 s
$m_{B}=m_{D}$	1.33 kg
$I_{B}=I_{D}$	$I_{3}$ [0.0017 0.0020 0.0022] kg m^2^
${\bar{r}}_{D/I}^{I}$	[−4069503 2861786 4483608] m
${\bar{v}}_{D/I}^{I}$	[−5114 −5691 −1000] m/s
${\bar{\omega }}_{D/I}^{D}$	[−1.0891 0.0326 0.5525] rad/s
$q_{D/I}$	[0.4559 0.6396 0.0356 −0.6179]
${\bar{r}}_{B/D}^{B}$	[10r 10r 10r] m
${\bar{v}}_{B/D}^{B}$	[r r r] m/s
${\bar{\omega }}_{B/D}^{B}$	[r r r] rad/s
$q_{B/D}$	[r r r r]

## Data Availability

The data presented in this study are available on request from the corresponding author.
